# Association between protein intake and sources in mid-pregnancy and the risk of gestational diabetes mellitus

**DOI:** 10.1186/s12884-025-07335-3

**Published:** 2025-03-06

**Authors:** Rui Wang, Xingyi Jin, Jian Zhu, Xiaocheng Li, Jian Chen, Chunyan Yuan, Xiaoli Wang, Yufeng Zheng, Shaokang Wang, Guiju Sun

**Affiliations:** 1 Key Laboratory of Environmental Medicine and Engineering of Ministry of Education, Department of Nutrition and Food Hygiene, School of Public Health, Southeast University, Nanjing, 210009 China; 2 Danyang Maternal and Child Health Hospital, Danyang, 212300 Zhenjiang China; 3 Nanjing Center for Disease Control and Prevention, Nanjing, 210003 China; 4 Department of Gynaecology and Obstetrics, Zhongda Hospital, Southeast University, 210009 Nanjing, China; 5 Xinjiang Uygur Autonomous Region Maternal and Child Health Hospital, 830000 Wulumuqi, China; 6 Aksu Region Maternal and Child Health Hospital, Aksu, 844000 China; 7 Clinical Medical Research Center for Plateau Gastroenterological Disease of Xizang Autonomous Region, and School of Medicine, Xizang Minzu University, Xianyang, 712082 China

**Keywords:** Dietary protein, Gestational diabetes mellitus, Substitution analysis, Prenatal nutrition, Pregnancy diet

## Abstract

**Objectives:**

This study aimed to investigate the relationship between dietary protein intake and sources in the second trimester of pregnancy and the risk of gestational diabetes mellitus (GDM) and to further investigate the effects of total protein and animal protein intake on the risk of GDM.

**Methods:**

A case-control study was conducted, which involved 947 pregnant women in the second trimester from three hospitals in Jiangsu, China. Dietary intake was assessed using a 3-day 24-hour dietary recall and a food frequency questionnaire. Two models (leave-one-out and partition models) in nutritional epidemiology were used for substitution analysis, and logistic regression was performed to explore the relationships, adjusting for multiple confounding factors.

**Results:**

After adjusting for confounding factors, total protein intake was negatively correlated with GDM risk (OR [95% CI], 0.10 [0.04–0.27]; *P*<0.001). Animal protein also negatively correlated with GDM risk, but this became insignificant when total calorie, carbohydrate and fat intake were added as covariates to the analysis (0.68 [0.34–1.34]; *P* = 0.263). No association was found between plant protein and GDM(1.04 [0.69–1.58]; *P* = 0.852). Replacing carbohydrates with an equal energy ratio(5% of total energy intake) of total protein, animal protein and plant protein respectively reduced the risk of GDM by 45%, 46% and 51%.

**Conclusions:**

The intake of total protein and animal protein, especially eggs, dairy products, and fish, can reduce the risk of GDM while consuming unprocessed red meat increases the risk. There is no significant association between the intakes of plant protein, processed meat, and poultry meat and the occurrence of GDM. The results of this study are expected to provide a basis for precise nutritional education, health guidance during pregnancy, and early prevention of GDM.

**Supplementary Information:**

The online version contains supplementary material available at 10.1186/s12884-025-07335-3.

## Introduction

Gestational diabetes mellitus (GDM) refers to diabetes or abnormal glucose metabolism diagnosed for the first time during pregnancy. It is one of the common endocrine disorders during pregnancy and is usually detected between 24 and 28 weeks of gestation. In most women, blood glucose returns to normal after delivery [1]. Although GDM may resolve spontaneously after childbirth, its impact on the health of both the mother and the offspring cannot be ignored, especially the increased risk of developing type 2 diabetes mellitus (T2DM) in the mother in the future and the short term and long term health effects on the offspring [2–6]. GDM and T2DM share similarities in pathogenesis, closely related to insulin resistance and insufficient pancreatic β-cell function. During pregnancy, hormonal changes, such as human placental lactogen, estrogen, and progesterone, promote insulin resistance, and the secretion of these hormones by the placenta gradually increases in the late stage of pregnancy [7, 8]. This insulin resistance leads to an increased demand for insulin in pregnant women. Under normal circumstances, the pancreas can secrete enough insulin to meet this demand. In some situations, pancreatic β-cells may not adjust to increasing insulin demand, leading to high blood glucose and GDM [9].

The main influencing factors of GDM include genetic factors, obesity and weight management, age during pregnancy, and diet and nutrition [10–12]. Dietary consumption, as a changeable factor, is strongly linked to GDM. Obesity and excessive weight gain, for example, can aggravate insulin resistance and raise the risk of developing GDM [13, 14]. Dietary fiber, monounsaturated fatty acids (such as those in olive oil and nuts), and polyunsaturated fatty acids (such as omega-3 fatty acids in fish oil) all assist to increase insulin sensitivity and prevent GDM [15, 16].

Macronutrients, including carbohydrates and fats, have previously been evaluated about GDM risk. However, the link with proteins remains uncertain. During pregnancy, particularly in the second trimester, the body experiences major physiological changes, increasing the demand for nutrients to support the fetus’s growth and development. Protein, in particular, plays a crucial role during this period and thus requires special attention. In the second trimester, the fetus enters a stage of rapid growth. Protein is the foundation for the development of fetal tissues and organs [17]. For instance, it is essential for the formation of the fetal muscle, bone, and connective tissues. Adequate protein intake can ensure that the fetus develops properly, promoting healthy muscle mass and skeletal structure [18, 19].

Although protein intake is essential during pregnancy, observational studies have shown inconsistent results regarding the relationship between total protein intake or different types and sources of protein and GDM. Some studies have demonstrated that adequate consumption of high-quality proteins (such as dairy products, seafood, and lean meat) aids in blood glucose control and reduces the incidence of T2DM [20, 21]. In contrast, high protein consumption may impair insulin action and cause blood glucose swings [22]. Wei Bao et al. [23] examined data from a large cohort study in the United States and discovered that prepregnancy animal protein intake, particularly red meat, was significantly positively correlated with the risk of GDM, whereas plant protein intake, particularly nuts, was negatively correlated with the risk. However, another study of the US population found that higher animal protein intake in the diet did not raise the incidence of GDM [24].

The divergent results of previous studies may be explained by study design, racial and ethnic differences in the populations, confounding factors, and differences in sample sizes. Given the scarcity of research on the relationship between protein intake and the risk of GDM in East Asia, as well as the conflicting findings of previous studies, the purpose of this case-control study is to investigate the relationship between dietary protein intake (total protein, animal protein, and plant protein) and the risk of GDM in pregnant women in Jiangsu, China. We also used substitution analysis to evaluate the impact of replacing carbohydrates with proteins and the mutual substitution of plant and animal proteins on the risk of GDM. On the basis of the results of substitution analysis, the proteins that have an impact on the risk of GDM were further analyzed according to different food sources.

## Subjects and methods

### Study subjects

This case-control study was carried out in the Danyang Maternal and Child Health Hospital, Nanjing Maternal and Child Health Hospital and Nanjing Tongren Hospital in Jiangsu Province, China. We enrolled pregnant women in the second trimester and trained investigators to conduct one-on-one information surveys before GDM screening at 20–28 weeks of gestation. The inclusion criteria for the study population were as follows: pregnant women diagnosed with singleton pregnancy within the first 18 weeks of pregnancy, voluntarily participating in the study with informed consent, and able to accurately understand and answer the questionnaire. The exclusion criteria were as follows: lack of fasting plasma glucose (FPG) or 75 g oral glucose tolerance test (OGTT) data; pregnant women with multiple pregnancies under 18 years old; pregnant women previously diagnosed with syphilis, AIDS, anemia, cardiopulmonary diseases, metabolic disorders, or psychological disorders; pregnant women with polycythemia vera. All participants signed a written informed consent form. 64 participants were disqualified from the study because they were unable to finish the food questionnaire. From September 2021 to November 2023, a total of 974 pregnant women voluntarily participated in this case-control study, among whom 453 were diagnosed with GDM. The pregnant women with normal blood glucose were selected as the control group in this study according to the difference of gestational weeks no more than 7 days. This study was approved by the School of Public Health of Southeast University and the Ethics Review Committee of the Nanjing Center for Disease Control and Prevention (PJ2020-A001-01).

#### Dietary intake assessment

Pregnant women participating in the project at the Danyang Maternal and Child Health Hospital during the second trimester were required to bring a three-day diet record to the hospital for routine examination. Dietitians used the 3-day 24-hour dietary recall method to provide individual nutritional assessments for each woman in the second trimester of pregnancy(supplementary Table 1). Pregnant women recruited in the Nanjing Maternal and Child Health Hospital and Nanjing Tongren Hospital were required to recall the dietary frequency of the past month before the OGTT and fill in a specifically designed food frequency questionnaire (supplementary Table 2). The dietary frequency questionnaire was based on the standardized Cronbach’s coefficient of 0.764, the KMO test statistic of 0.632, and Bartlett’s sphericity test χ^2^ = 1340.408 (*P* < 0.001), indicating good reliability and validity. The total intake of a certain nutrient was calculated by summing the product of the intake of each food containing that nutrient and the content of that nutrient in the corresponding food. The study needed to determine the average daily dietary intake, the intakes of the three primary energy supplying nutrients (carbohydrates, proteins, and fats), plant protein, animal protein, and the percentage of energy provided by these nutrients.

Researchers determined the average content of various nutrients in a certain food through the Food Nutrition Composition Query Platform. The Food Nutrition Composition Query Platform was jointly launched by the National Institute for Nutrition and Health of the Chinese Center for Disease Control and Prevention and the Chinese Nutrition Society. The data on the platform mainly came from the Chinese Food Composition Table. About 1300 common foods were screened and classified into 40 categories, including cereals and products, tubers, starches and products, dried legumes and products, fruits and products, mushrooms, nuts, livestock meat and products, infant foods, snacks, and beverages. The food composition query platform provided 31 food nutrient components for readers to refer to and updated the data in real-time. In addition, it also offered three nutritional tools (Nutrition Assistant, My Plate, and Nutrition Label Query) for readers to use online.

#### GDM diagnostic criteria

According to the IADPSG criteria: pregnant women undergo a 75 g OGTT with glucose thresholds of 5.1, 10.0, and 8.5 mmol/L for fasting, 1 h, and 2 h after oral glucose intake, respectively, and a diagnosis of GDM is made when the glucose value reaches or exceeds the above criteria at any of these time points [25].

### Statistical analysis methods

This analysis mainly focuses on the impacts of protein replacing carbohydrates and the mutual substitution of different protein sources on the risk of GDM. Since different food frequency survey methods were used in the population, the nutrient intake in all models was expressed as energy density (the energy provided by a certain nutrient / total energy × 100%) to reduce the error in nutrient intake among individuals caused by different food frequency survey methods.

Two commonly used models in nutritional epidemiology were used to adjust for total energy in the substitution analysis: the leave-one-out and partition models. Previous studies have shown that substitution analysis involving a single exposure and a single surrogate component has high accuracy [26]. The leave-one-out model can be expressed as GDM risk = a1 Protein energy + a2 Fat energy + a3 Total energy = a1 Protein energy + a2 Fat + a3 (Protein energy + Fat energy + Carbohydrates energy). When protein and fat intake are considered in the model, then the total energy only includes the carbohydrate component. Therefore, when the total energy intake is added to the model, the coefficient of total energy does not directly reflect the effect of total energy on disease risk. It represents the impact of carbohydrate intake on disease risk because fat and protein intake has been independently considered in the total energy. In the leave-one-out model, the substitution effect of protein for carbohydrates can be directly represented by (a1 + a3)-a3 = a1. Correspondingly, when protein is divided into plant protein and animal protein according to food sources, carbohydrates are added to the model, and animal protein is excluded from the model, the coefficient of plant protein can represent the substitution effect of plant protein for animal protein.

The partition model can be expressed as GDM risk = b1 Protein energy + b2 Fat energy + b3 Carbohydrates energy. In the partition model, the parameter of the substitution effect of protein for carbohydrates is (b1 - b3). It should be noted that when calculating the variance of the substitution effect parameter in the partition model, the covariance between the two coefficients needs to be obtained, and the variance estimation formula is: var(b1 - b3) = var(b1) + var(b3) − 2cov(b1, b3) [27]. A key prerequisite assumption for any substitution analysis is that each person’s total consumption of different nutrients or foods being substituted is known and limited to a certain level. Otherwise, the substitution effect cannot be well defined, which may make the results difficult to interpret. Substitution analysis of macronutrients can satisfy the above conditions well, but we must be cautious about substitution analysis of other nutrients or foods.

Based on known associations with GDM risk and potential impacts on metabolic health, considering the impact of potential confounding factors on the results, in the logistic regression model, the results adjusted for age and BMI, as well as further adjusted for family history of diabetes (yes/no), adverse pregnancy and childbirth history (yes/no), regular physical activity (yes/no), secondhand smoke exposure (yes/no), and alcohol consumption (yes/no) were provided. BMI was calculated as weight (kg) divided by the square of height (m). During the survey, it was found that the probabilities of pregnant women actively smoking and drinking after pregnancy were extremely low. Therefore, we additionally investigated the secondhand smoke exposure of pregnant women and extended the time span of alcohol consumption to six months before pregnancy. Three or more sessions of moderate-to-intense physical exercise per week (lasting at least 25 min each) are considered regular physical activity. Adverse pregnancy and childbirth history included miscarriage, preterm birth, stillbirth, gestational hypertension, GDM, uterine scar, and other delivery complications.

According to the distribution types of participant characteristic information variables, the mean ± standard deviation or median (upper and lower quartiles) was used for expression. Logistic regression was used to explore the impacts of replacing carbohydrates with proteins from different food sources and the mutual substitution of proteins from different food sources on the relationship with GDM, and the corresponding odds ratios (ORs) and 95% confidence intervals (CIs) were calculated. All the above statistical analyses were performed using SPSS 25.0 software and R 4.3.1 software, with two sided tests and α = 0.05.

## Results

We divided the continuous protein intake of 974 people into quartiles and counted their essential characteristics (Table 1). As shown in the table, the group with higher total protein intake was more likely to have no secondhand smoke exposure and more likely to engage in regular physical activity weekly. After grouping by animal protein intake, the same results were obtained. In addition, there was a specific relationship between animal protein intake and alcohol consumption. The groups with the lowest and highest animal protein intakes had a higher probability of alcohol consumption. After comparing the GDM prevalence rates of each group, it was found that the P50 - P75 groups of total protein and animal protein had a lower GDM prevalence rate than other groups.

**Table 1:** Baseline characteristics at the quartile of 947 participants during pregnancy

	Total protein				Vegetable protein				Animal protein			
	≤P25	P25~P50	P50~P75	> P75	≤P25	P25~P50	P50~P75	> P75	≤P25	P25~P50	P50~P75	> P75
Participants (n)	237	237	237	236	237	237	237	236	237	237	237	236
Age (years)	29.40 ± 4.14	29.47 ± 4.01	29.73 ± 3.85	30.08 ± 4.20	29.91 ± 4.20	29.83 ± 3.96	29.69 ± 3.69	29.24 ± 4.33	29.39 ± 4.03	29.51 ± 4.15	29.66 ± 3.75	30.12 ± 4.26
Family history of diabetes (%)	21 (8.9)	38 (16.0)	24 (10.1)	30 (12.7)	27 (11.4)	25 (10.5)	28 (11.8)	33 (14.0)	29 (12.2)	29 (12.2)	28 (11.8)	27 (11.4)
History of adverse pregnancy (%)	78 (32.9)	77 (32.5)	54 (22.8)	73 (30.9)	72 (30.4)	63 (26.6)	70 (29.5)	77 (32.6)	84 (35.4)	62 (26.2)	66 (27.8)	70 (29.7)
Secondhand smoke exposure (%)	70 (29.5)	63 (26.6)	45 (19.0)	43 (18.2)	59 (24.9)	45 (19.0)	62 (26.2)	55 (23.3)	68 (28.7)	63 (26.6)	42 (17.7)	48 (20.3)
Alcohol exposure (%)	15 (6.3)	14 (5.9)	6 (2.5)	15 (6.4)	14 (5.9)	11 (4.6)	12 (5.1)	13 (5.5)	20 (8.4)	9 (3.8)	5 (2.1)	16 (6.8)
Physical activity (%)	108 (45.6)	151 (63.7)	178 (75.1)	185 (78.4)	151 (63.7)	170 (71.7)	154 (65.0)	147 (62.3)	117 (49.4)	143 (60.3)	180 (75.9)	182 (77.1)
BMI (kg/m^2^)	22.21 ± 3.32	22.29 ± 3.47	22.29 ± 3.30	21.93 ± 3.22	22.11 ± 3.47	22.42 ± 3.23	22.11 ± 3.14	22.1 ± 3.47	22.22 ± 3.32	22.20 ± 3.19	22.18 ± 3.63	22.13 ± 3.18
Total calories (kcal)	1667.44 ± 555.70	1768.21 ± 438.05	1852.29 ± 494.93	1996.71 ± 626.58	1919.03 ± 681.48	1810.90 ± 461.71	1824.12 ± 517.95	1729.47 ± 481.57	1666.16 ± 558.16	1775.39 ± 466.34	1838.98 ± 410.64	2004.15 ± 662.65
Carbohydrate (%E)	61.28 ± 6.84	56.49 ± 4.68	53.59 ± 4.45	48.91 ± 6.06	55.60 ± 7.90	55.20 ± 6.62	54.89 ± 6.17	54.60 ± 7.83	59.41 ± 7.27	56.40 ± 6.15	54.32 ± 4.89	50.15 ± 6.80
Total fat (%E)	22.91 ± 6.53	24.47 ± 4.51	25.47 ± 4.38	26.49 ± 4.84	23.36 ± 5.30	24.77 ± 4.61	25.38 ± 4.74	25.84 ± 6.12	24.21 ± 6.46	24.52 ± 5.24	24.94 ± 4.05	25.68 ± 5.09
GDM(%)	138 (58.2)	130 (54.9)	83 (35.0)	102 (43.2)	110 (46.4)	111 (46.8)	105 (44.3)	127 (53.8)	147 (62.0)	114 (48.1)	79 (33.3)	113 (47.9)

P25, 25th percentile; P50, 50th percentile; P75, 75th percentile; Values are means (SD) unless otherwise specified; BMI, Body mass index; GDM, Gestational diabetes mellitus; %E, % of energy; Significant comparisons in total protein groups include secondhand smoke exposure, physical activity, total calories, carbohydrate, total fat, GDM; Significant comparisons in vegetable protein groups include total calories, total fat; Significant comparisons in animal protein groups include secondhand smoke exposure, alcohol exposure, physical activity, total calories carbohydrate, total fat, GDM

The mean of protein energy density was 20.09%, the mean of animal protein energy density was 12.38%, and the mean of plant protein energy density was 7.71%. In the lowest and highest quartile groups, the median total protein intakes were 16.14% and 23.98%, respectively; the animal protein intakes were 7.69% and 17.01%, and the plant protein intakes were 5.58% and 9.83%, respectively. After adjusting for age, BMI, family history of diabetes, adverse pregnancy and childbirth history, regular physical activity, secondhand smoke exposure, and alcohol consumption, the total protein intake and animal protein intake were negatively correlated with the risk of GDM. The OR (95%CI) for comparing the highest and lowest quintiles of total protein intake was 0.53 (0.36-0.79), and that for animal protein was 0.57 (0.39–0.84). However, when total calorie intake, carbohydrate intake, and fat intake were added as covariates to the equation, the association between animal protein and GDM was not significant, and the total protein intake was still negatively correlated with the risk of GDM, with an OR(95%CI) of 0.10 (0.04–0.27). After adjusting for different confounding factors, no association was found between plant protein and the risk of GDM (Table 2).

**Table 2:** OR (95% CIs) of GDM based on preconception dietary protein intake quartile

	≤P25		P25 ~ P50		P50~P75		> P75		*P*-value^a^
Total protein									
Median intake(%E)	16.14		19.04		20.93		23.98		
ORs (95% CI)*	1		0.86 (0.60–1.25)		0.37 (0.25–0.54)		0.51 (0.35–0.74)		<0.001
ORs (95% CI)**	1		0.87 (0.59–1.27)		0.40 (0.27–0.59)		0.53 (0.36–0.79)		0.002
ORs (95% CI)***	1		0.46 (0.28–0.77)		0.13 (0.07–0.26)		0.10 (0.04–0.27)		<0.001
Animal protein									
Median intake(%E)	7.69		11.16		13.36		17.01		
ORs (95% CI)*	1		0.56 (0.38–0.80)		0.29 (0.20–0.43)		0.53 (0.36–0.77)		<0.001
ORs (95% CI)**	1		0.59 (0.40–0.86)		0.32 (0.22–0.48)		0.57 (0.39–0.84)		0.005
ORs (95% CI)***	1		0.60 (0.38–0.94)		0.34 (0.20–0.56)		0.68 (0.34–1.34)		0.263
Vegetable protein									
Median intake(%E)	5.58		7.01		8.23		9.83		
ORs (95% CI)*	1		1.02 (0.71–1.47)		0.93 (0.65–1.34)		1.42 (0.98–2.05)		0.062
ORs (95% CI)**	1		1.07 (0.74–1.56)		0.94 (0.65–1.37)		1.40 (0.96–2.04)		0.078
ORs (95% CI)***	1		0.86 (0.57–1.29)		0.66 (0.43–0.99)		1.04 (0.69–1.58)		0.852

OR, Odds ratio; CI, Confidence interval; a, Significance of the highest percentile compared with baseline; *, Logistic regression with adjustment for age and BMI; **, Logistic regression with adjustment for age, BMI, family history of diabetes, history of adverse pregnancy, secondhand smoke exposure, alcohol exposure, physical activity; ***, On the basis of the previous adjustment of total calories, carbohydrate, total fat

The results of the impacts of replacing carbohydrates with total protein and proteins from different food sources with an equal energy ratio (5% of total energy intake) on the risk of GDM are shown in Table 3. In the leave-one-out model, replacing carbohydrates with protein was associated with a 45% reduction in the risk of GDM (OR [95% CI], 0.55 [0.44–0.69]; *P* < 0.001). After replacing carbohydrates with animal protein, the risk was reduced by 46% (0.54 [0.43–0.69]; *P* < 0.001). Replacing carbohydrates with plant protein reduced the risk by 51% (0.49 [0.30–0.79]; *P* = 0.004). In the partition model, replacing dietary protein with an equal energy of carbohydrates during pregnancy was still associated with a reduced risk of GDM. This association was not significantly changed by age, BMI, adverse pregnancy and childbirth history, family history of diabetes, or physical activity. We additionally provided the results after adjusting for total energy intake. After replacing isoenergetic carbohydrates with total protein, animal protein, and plant protein, the risk of pregnant women developing GDM was reduced by 45% (0.55 [0.42–0.68]; *P* < 0.001), 46% (0.54 [0.41–0.67]; *P* < 0.001), and 51% (0.49 [0.25–0.72]; *P* = 0.004). The results were the same as those obtained in the leave-one-out model. Unfortunately, in both models, no correlation was found between plant protein intake and the risk of GDM, and the substitution effect between plant protein and animal protein was also not significant.

**Table 3:** ORs (95% CIs) of GDM associated with increases in 5% of energy from types of protein

	ORs (95% CIs)*	*P*-value	ORs (95% CIs)**	*P*-value	ORs (95% CIs)***	*P*-value
**Leave-one-out model**						
Substitution for carbohydrate intake						
Total protein	0.52(0.41–0.65)	<0.001	0.55(0.44–0.69)	<0.001	--	--
Animal protein	0.51(0.41–0.64)	<0.001	0.54(0.43–0.69)	<0.001	--	--
Vegetable protein	0.47(0.29–0.76)	0.002	0.49(0.30–0.79)	0.004	--	--
Substitution for vegetable protein intake						
Animal protein	1.09(0.75–1.57)	0.650	1.11(0.76–1.61)	0.587	--	--
Substitution for animal protein intake						
Vegetable protein	0.92(0.64–1.33)	0.650	0.90(0.62–1.31)	0.587	--	--
**Partition model**						
Substitution for carbohydrate intake						
Total protein	0.61(0.48–0.74)	<0.001	0.64(0.49–0.78)	<0.001	0.55(0.42–0.68)	<0.001
Animal protein	0.60(0.47–0.73)	<0.001	0.62(0.48–0.76)	<0.001	0.54(0.41–0.67)	<0.001
Vegetable protein	0.50(0.27–0.73)	0.003	0.51(0.27–0.74)	0.004	0.49(0.25–0.72)	0.004
Substitution for vegetable protein intake						
Animal protein	1.19(0.77–1.62)	0.325	1.22(0.78–1.66)	0.274	1.11(0.70–1.52)	0.587
Substitution for animal protein intake						
Vegetable protein	0.84(0.54–1.13)	0.325	0.82(0.52–1.11)	0.274	0.90(0.57–1.24)	0.587

OR, Odds ratio; CI, Confidence interval; *, The model was adjusted for age and BMI; **,The model was adjusted for age, BMI, family history of diabetes, history of adverse pregnancy, secondhand smoke exposure, alcohol exposure, physical activity; ***, the previous adjustment plus total calories

To further explore the impacts of total protein and animal protein intakes on the risk of GDM, we compared the dietary data of 755 pregnant women who provided FFQ results between the GDM group and the non-GDM group (Table 4) and then performed logistic regression analysis. The results are shown in Table 5. The results of adjusting for different confounding factors all showed that unprocessed red meat consumption during pregnancy increased the risk of GDM (OR [95% CI], 1.04 [1.03–1.05], *P* < 0.001), and dairy product consumption reduced the risk of GDM (OR [95% CI], 0.99 [0.98–0.99], *P* < 0.001). Before adjusting for dietary related factors, an increase in fish intake was negatively correlated with the risk of GDM (OR [95% CI], 0.99[0.98-1.00], *P* = 0.024).

**Table 4:** Animal protein intake from FFQ data of 755 participants

	GDM(*n* = 374)	Without GDM(*n* = 381)	*P*-value
Energy (kcal)	2050.7 ± 564.15	1838 ± 373.86	<0.001
Carbohydrate (%E)	53.79 ± 7.54	55.66 ± 4.99	<0.001
Total fat (%E)	26.01 ± 5.01	23.25 ± 3.57	<0.001
Total protein (%E)	20.20 ± 3.65	21.09 ± 2.54	<0.001
Vegetable protein (g)	7.72 ± 2.36	7.46 ± 1.68	0.082
Animal protein (g)	12.48 ± 4.78	13.63 ± 3.00	<0.001
Unprocessed red meat (g)	76.96 ± 65.75	45.75 ± 24.12	<0.001
Processed red meat (g)	1.71 ± 3.84	1.61 ± 3.15	0.702
Poultry (g)	22.25 ± 29.24	21.47 ± 18.45	0.660
Fish (g)	47.77 ± 45.7	61.61 ± 34.7	<0.001
Dairy products (g)	269.59 ± 118.94	299.02 ± 149.33	0.003
Eggs (g)	50.83 ± 25.39	50.88 ± 19.49	0.972

GDM, Gestational diabetes mellitus; %E, % of energy;

**Table 5:** ORs (95% CIs) for GDM according to logistic regression results from 755 participants

	ORs (95% CIs)*	*P*-value	ORs (95% CIs)**	*P*-value	ORs (95% CIs)***	*P*-value
Unprocessed red meat	1.02(1.01–1.03)	<0.001	1.02(1.02–1.03)	<0.001	1.04(1.03–1.05)	<0.001
Processed red meat	1.00(0.96–1.04)	0.912	1.01(0.96–1.05)	0.832	0.99(0.94–1.04)	0.689
Poultry	1.00(0.99–1.01)	0.476	1.00(0.99–1.01)	0.509	1.00(0.99–1.01)	0.761
Fish	0.99(0.98–0.99)	<0.001	0.99(0.98–0.99)	<0.001	1.00(0.99–1.01)	0.179
Dairy products	0.99(0.99-1.00)	0.006	0.99(0.99–1.01)	0.020	0.99(0.98–0.99)	<0.001
Eggs	0.99(0.99–1.01)	0.396	1.00(0.99–1.01)	0.700	0.99(0.98-1.00)	0.024

OR, Odds ratio; CI, Confidence interval; *, The model was adjusted for age and BMI; **,The model was adjusted for age, BMI, family history of diabetes, history of adverse pregnancy, secondhand smoke exposure, alcohol exposure, physical activity; ***, the previous adjustment plus total calories

## Discussion

### Total protein intake and GDM

Our study results showed that increasing protein intake can reduce the risk of GDM, and replacing carbohydrates with an equal amount of protein (5% of total energy) reduced the risk of GDM by 45%. The risk reduction of GDM associated with protein intake may be related to the multiple mechanisms by which a high-protein diet affects blood glucose control. For example, protein intake is closely related to weight management, and maintaining a healthy weight during pregnancy is crucial for preventing GDM. A high-protein diet often helps increase satiety, control appetite, reduce overeating, and delay gastric emptying, thereby delaying sugar absorption and avoiding rapid postprandial blood glucose elevation, which is beneficial for weight control [28, 29]. The composition of the gut microbiota is closely related to the occurrence of diabetes, and protein may play an essential role in regulating the gut microbiota. A high-protein diet may improve glucose metabolism and reduce the occurrence of insulin resistance by regulating the diversity of the gut microbiota and promoting the growth of beneficial bacteria [30]. The occurrence of GDM may also be related to the function of the immune system, especially immune regulation during pregnancy. Protein is a key nutrient required for immune responses and anti-inflammatory effects, and sufficient protein intake may help maintain normal immune function. Some amino acids in protein (such as glutamine) help reduce inflammation [31], which may reduce the risk of GDM by improving insulin action.

A high-protein diet may help reduce fat accumulation, especially abdominal fat deposition, which is crucial for preventing insulin resistance. Protein intake may directly affect insulin secretion. The digestion products of protein(amino acids) can stimulate insulin secretion and help maintain blood glucose stability [32]. Protein also has a particular impact on insulin sensitivity. For example, Tettamanzi [33] conducted a 21-day randomized controlled inpatient crossover feeding trial in 20 insulin-resistant obese women to evaluate the degree of effect of Mediterranean and high-protein diets on metabolic parameters. The results showed that compared with the Mediterranean diet, the high-protein diet was more effective in reducing insulin resistance and improving blood glucose variability, further illustrating that a high-protein diet can improve insulin efficacy.

The risk of GDM is decreased by these effects, which are cumulative. It is still up for debate, nevertheless, whether certain protein sources are consistently linked to the risk of GDM throughout pregnancy.

### Animal protein, red meat and GDM

In the study by Wei Bao, animal protein intake was found to increase the risk of GDM [23], which is different from the conclusion of this study. The main reason may be that their study population mainly consisted of white American women. Therefore, the generalizability of the study results to the Han population may be limited. The 2021 Chinese Dietary Science Research Report pointed out that 40.5% of the dietary protein of Chinese urban and rural residents comes from animal foods [34]. Animal protein is an important source of protein for the human body. It contains various essential amino acids the human body requires, which are crucial for maintaining the normal physiological functions of pregnant women and fetuses. However, the relationship between animal protein and GDM is complex and not simply positive or negative. On the one hand, an appropriate animal protein intake during pregnancy is beneficial. It helps fetal growth and development, such as supporting the construction of fetal tissues and organs, especially the development of muscles and bones [18].

On the other hand, although animal protein contains a complete amino acid spectrum, excessive intake of animal protein, especially red meat and processed meat, may be associated with an increased risk of chronic diseases (such as insulin resistance and obesity) and death [23, 35–37]. In this study, red meat consumption was significantly associated with an increased risk of GDM, which is consistent with previous research results [23, 38]. Red meat usually contains relatively high saturated fat. As mentioned above, excessive intake of saturated fat leads to fat accumulation in the body, especially abdominal fat deposition, which causes insulin resistance and reduces the sensitivity of body cells to insulin, significantly increasing the possibility of pregnant women developing GDM [13, 14]. Additionally, a study of metabolomics recently demonstrated that Elevated levels of branched-chain amino acids (e.g., isoleucine, leucine, and valine) are associated with an increased risk for diabetes [39]. Branched-chain amino acids can affect insulin signaling by activating the mammalian target of the rapamycin signaling pathway [40]. These metabolite changes may reflect early metabolic features of insulin resistance and β-cell dysfunction. The FIGO Nutrient Checklist formulated by the Chinese University of Hong Kong team recommends consuming meat or chicken 2–3 times a week [41]. The Mediterranean diet pattern has been proven to reduce the risk of diabetes, and this pattern shows that a red meat intake frequency of ≤ 2 times a week is the most ideal [42].

According to the results of our study, consuming dairy products can reduce the risk of GDM. In previous studies, dairy product consumption is usually associated with a lower risk of T2DM [43]. Dairy product intake can prevent GDM through favorable effects on known risk factors or precursors of diseases such as body weight, hypertension, and abnormal glucose homeostasis [44–46]. After adjusting for energy intake, we also observed that egg intake can reduce the risk of GDM. Although some studies have shown that adhering to a cholesterol rich diet pattern in early pregnancy can increase the risk of GDM [47], in our study, the daily egg intake of pregnant women is approximately 50 g, equivalent to one egg. The cholesterol content does not reach the pathogenic intake. The study by Qiu C claims that only when the egg intake is higher than seven eggs per week can the cholesterol intake from eggs by pregnant women be related to the risk of GDM [48].

### Plant protein, white meat and GDM

In this study, no significant association was found between plant protein and GDM, which is consistent with the previous research results of Zhang [49]. However, some animal experiments and population studies have found that plantbased proteins (such as beans, nuts, and seeds) are more beneficial for improving insulin sensitivity compared with animal proteins (such as red meat and processed meat), and may reduce the risk of GDM by reducing the inflammatory response [50–52]. Different regions have different definitions of the sources of plant protein. In this study, plant protein mainly comes from grains, beans, and nuts. The low consumption frequency of beans and nuts in Jiangsu, China, demographic differences such as ethnicity, different dietary characteristics in different regions, and different food cooking methods may all be the reasons for the differences between this study and previous studies. In addition, due to the high correlation between nutrients and proteins in familiar food sources, we cannot rule out the possibility of over adjustment, which may lead to an underestimation of the true association between plant protein intake and the risk of GDM.

Compared with red meat, white meat usually has lower fat content, higher unsaturated fatty acid content, and easier digestion and absorption, and often occupies an essential position in healthy diet recommendations. In this study and the study by Meinilä, although no association was found between poultry meat and GDM [53], before adjusting for dietary related factors, an increase in fish intake was negatively correlated with the occurrence of GDM. A study from Iceland also showed that a fish intake of < 2 times per week is a dietary risk factor associated with GDM [54]. Although the association between fish and GDM was no longer significant after adjusting for energy intake, it still indicates that the intake of white meat does not increase the risk of GDM.

The adverse impacts of GDM on the mother’s and the fetus’s health must be disregarded, even if the majority of instances may go away on their own after delivery. The HAPO FUS study found that among patients with GDM diagnosed according to the IADPSG criteria, 52.2% had abnormal glucose metabolism (including impaired fasting glucose, impaired glucose tolerance, and T2DM) 10–14 years after childbirth. In comparison, the proportion in the control group was 20.1%. The OR for prediabetes was 3.44 (95%CI: 2.85–4.41), and the OR for T2DM was 5.44 (95%CI: 3.68–8.08). Moreover, the glucose and lipid metabolism and obesity outcomes of the mother and offspring after childbirth are also continuously and linearly correlated with the mother’s blood glucose during pregnancy [55]. That is, the higher the mother’s blood glucose during pregnancy, the greater the possibility of abnormal glucose and lipid metabolism in the two generations of the mother and child after childbirth, and the higher the risk of obesity in the offspring.

The HAPO study and the HAPO FUS study remind us that intrauterine blood glucose is a window into the future metabolic status of the two generations of the mother and child [5, 55]. Even a mildly elevated high glucose environment in the uterus has a long term and profound impact on the two generations of the mother and child. Currently, the common treatment for gestational diabetes mellitus is oral hypoglycemic agents, such as metformin [56]. However, Studies have shown that intrauterine exposure to antidiabetic drugs has longitudinal growth effects [57]. Our study’s findings can inform public health policies. Promote consumption of beneficial proteins like eggs, dairy, and fish, and limit unprocessed red meat. Standardize dietary assessment tools. Emphasize physical activity and smoking cessation. These steps can help reduce GDM incidence and improve outcomes for pregnant women and their offspring.

The advantages of this study are as follows: It is the first study to analyze the association between the dietary protein intake of pregnant women in Jiangsu, China, and the risk of GDM. At the same time, compared with previous dietary surveys based on the first trimester of pregnancy, the results of this study based on the second trimester dietary survey can better reflect the dietary habits during pregnancy. This study conducted a detailed classification of the sources of protein. It explored whether there is a correlation between different protein sources and the risk of GDM, providing help for precise dietary education and guidance during pregnancy.

Our study also had some limitations: (1) This is a case-control study, which can only explore the correlation between protein intake in the second trimester of pregnancy and the occurrence of GDM and explore the dietary influencing factors related to the occurrence of GDM but cannot make causal inferences. (2) This study used a dietary frequency intake questionnaire for a dietary survey, and pregnant women filled it out through recollection, and there is a recall bias. Previous studies have also shown that parturients cannot accurately assess dietary intake, and the assessment of dietary intake may have greater recall bias [58]. (3) The significant impacts of animal and plant protein on the incidence of GDM may be due to the simultaneous presence of other nutrients in protein rich foods. For example, cholesterol and saturated fat are simultaneously present in foods rich in animal protein. This study did not adjust for related dietary nutrients. Future research should adopt a prospective cohort or RCT design, use objective dietary assessment methods like food diaries or mobile apps, comprehensively assess all relevant nutrients with multivariate regression models, and explore interaction effects to overcome the limitations of previous studies and enhance understanding of the relationship between protein intake and GDM.

## Conclusions

In conclusion, consumption of animal and total proteins lowers the incidence of GDM. Among animal proteins, eating fish, dairy products, and eggs can lower the risk of developing GDM. However, eating unprocessed red meat raises the risk of developing GDM. There was no correlation discovered between the incidence of GDM and consumption of processed meat, plant protein, or poultry meat. To serve as a foundation for implementing precise nutritional education and health guidance during pregnancy and for the early prevention of GDM, this conclusion has to be further confirmed in large sample randomized prospective cohort studies conducted in China in the future.

## Electronic supplementary material

Below is the link to the electronic supplementary material.

Supplementary Material 1

## Data Availability

The datasets used and analysed during the current study available from the corresponding author on reasonable request.
