# Supplementary material for: Association between protein intake and sources in mid-pregnancy and the risk of gestational diabetes mellitus
Source: BMC Pregnancy Childbirth. 2025 Mar 6;25:240. doi: 10.1186/s12884-025-07335-3 (PMC11884067; doi:10.1186/s12884-025-07335-3)
Supplement: Supplementary file 1 — Supplementary Material 1 [file 12884_2025_7335_MOESM1_ESM.docx]

Supplementary table 1 Dietary questionnaire (3-day 24-hour method)

Name：____________ ID number：________________ Mobile phone number：_______________________ Current gestational week:______________

| **Number of meals** | **Day 1 (Date: ___________)** | **Day 3 (Date: ___________)** | **Day 3 (Date: ___________)** |
| --- | --- | --- | --- |
| The first meal |  |  |  |
| The second meal |  |  |  |
| The third meal |  |  |  |
| Snacks, beverages  and nutritional supplements |  |  |  |

Please fill in the following table with the name, ingredients and weight (accurate to grams) of the food you ate for three consecutive days.

Supplementary table 2 Food frequency questionnaire

| **Please recall whether you have eaten any of the following foods in the past month, and estimate the frequency and amount of each type of food.** | | | | | | |
| --- | --- | --- | --- | --- | --- | --- |
|  | | Whether to eat? If yes, tick √ | Frequency of consumption  (Fill in 1 item only) | | | Average weight per serving |
|  |  |  | Times/day | Times/week | Times/month |  |
| **Staple foods** | | | | | | |
| 1 | Rice and products (rice/rice flour, etc.) | □ | □/day | □/week | □/month | □□□g |
| 2 | Wheat flour and products (steamed bread/noodles, etc.) | □ | □/day | □/week | □/month | □□□g |
| 3 | Corn and its products (corn flour, corn ballast, etc.) (recorded by weight) | □ | □/day | □/week | □/month | □□□g |
| 4 | Other cereals and products (buckwheat/millet, etc.) (recorded by weight) | □ | □/day | □/week | □/month | □□□g |
| 5 | Beans (Mung beans/red beans/pinto beans, etc.) (recorded by weight) | □ | □/day | □/week | □/month | □□□g |
| 6 | Potatoes (potato/taro/sweet potato, etc.) (recorded by weight) | □ | □/day | □/week | □/month | □□□g |
| 7 | Fried pasta (fried dough sticks, pancakes, fried cakes, sesame balls, etc.) | □ | □/day | □/week | □/month | □□□g |
| 8 | Instant noodles | □ | □/day | □/week | □/month | □□□g |
| **Beans** | | | | | | |
| 9 | Dry beans (soybean/soybean/green bean/black bean, etc.) (dry weight) | □ | □/day | □/week | □/month | □□□g |
| 10 | Soybean milk, bean curd | □ | □/day | □/week | □/month | □□□g |
| 11 | Tofu | □ | □/day | □/week | □/month | □□□g |
| 12 | Other soybean products include shredded tofu, beancurd skin, thousand sheets, dried beancurd, beancurd bamboo, etc. | □ | □/day | □/week | □/month | □□□g |
| **Vegetables (recorded by edible weight)** | | | | | | |
| 13 | Fresh dark vegetables (raw weight) | □ | □/day | □/week | □/month | □□□g |
| 14 | Fresh light colored vegetables (raw weight) | □ | □/day | □/week | □/month | □□□g |
| 15 | Pickled vegetables (pickles, sauerkraut, pickles, etc.) | □ | □/day | □/week | □/month | □□□g |
| **Mushrooms and algae** | | | | | | |
| 16 | Mushrooms (Fresh weight) | □ | □/day | □/week | □/month | □□□g |
| 17 | Fungus, Tremella tremella, bamboo fungus, etc. | □ | □/day | □/week | □/month | □□□g |
| 18 | Kelp (Fresh weight) | □ | □/day | □/week | □/month | □□□g |
| 19 | Laver (dry weight) | □ | □/day | □/week | □/month | □□□g |
| 20 | Nori (dry weight) | □ | □/day | □/week | □/month | □□□g |
| **Fruits (recorded by edible weight)** | | | | | | |
| 21 | Fresh dark fruits | □ | □/day | □/week | □/month | □□□g |
| 22 | Fresh light colored fruit | □ | □/day | □/week | □/month | □□□g |
| 23 | Dried fruits such as dates, raisins, dried persimmons, bananas  Dried, dried apricot, etc. | □ | □/day | □/week | □/month | □□□g |
| **Milk** | | | | | | |
| 24 | Whole milk (milk powder fresh milk conversion ratio is 1:7) | □ | □/day | □/week | □/month | □□□g |
| 25 | Low fat, skim milk | □ | □/day | □/week | □/month | □□□g |
| 26 | Yogurt | □ | □/day | □/week | □/month | □□□g |
| 27 | Cheese | □ | □/day | □/week | □/month | □□□g |
| **Meat (recorded by edible weight)** | | | | | | |
| 28 | Pork | □ | □/day | □/week | □/month | □□□g |
| 29 | Poultry meat | □ | □/day | □/week | □/month | □□□g |
| 30 | Other animal meat (beef/mutton/donkey/horse/rabbit/dog meat, etc.) | □ | □/day | □/week | □/month | □□□g |
| 31 | Meat products (sausage/ham sausage/luncheon meat, etc.) | □ | □/day | □/week | □/month | □□□g |
| 32 | Animal viscera | □ | □/day | □/week | □/month | □□□g |
| **Aquatic products (recorded by edible weight)** | | | | | | |
| 33 | Sea fish (belt fish/yellow croaker/yellow croaker/flat fish, etc.) | □ | □/day | □/week | □/month | □□□g |
| 34 | Freshwater fish (carp/silver carp/bass/Wuchang fish) | □ | □/day | □/week | □/month | □□□g |
| 35 | Shrimp and crab | □ | □/day | □/week | □/month | □□□g |
| 36 | Molluscs (Squid/shellfish/snails/sea cucumbers) | □ | □/day | □/week | □/month | □□□g |
| **Eggs** | | | | | | |
| 37 | Fresh eggs | □ | □/day | □/week | □/month | □□□g |
| 38 | Salted eggs (salted duck egg/salted egg/salted goose egg) | □ | □/day | □/week | □/month | □□□g |
| 39 | Preserved eggs | □ | □/day | □/week | □/month | □□□g |
| **Others** | | | | | | |
| 40 | Snacks (such as bread, biscuits, pastries, potato chips, puffed food) | □ | □/day | □/week | □/month | □□□g |
| 41 | nuts (such as melon seeds, peanuts, walnuts, pistachios, hazelnuts and other edible (by weight) | □ | □/day | □/week | □/month | □□□g |
| 42 | Preserved fruit | □ | □/day | □/week | □/month | □□□g |
| 43 | Chocolate | □ | □/day | □/week | □/month | □□□g |
| 44 | Candy | □ | □/day | □/week | □/month | □□□g |
| 45 | Ice Cream/Popsicle | □ | □/day | □/week | □/month | □□□g |
| **Beverages** | | | | | | |
| 46 | Carbonated beverages | □ | □/day | □/week | □/month | □□□ml |
| 47 | Fresh fruit and vegetable juice | □ | □/day | □/week | □/month | □□□ml |
| 48 | Fruit and vegetable juice drinks | □ | □/day | □/week | □/month | □□□ml |
| 49 | Lactic acid Bacteria beverages | □ | □/day | □/week | □/month | □□□ml |
| 50 | Other sugary drinks | □ | □/day | □/week | □/month | □□□ml |
| **Drink** | | | | | | |
| 51 | Liquor | □ | □/day | □/week | □/month | □□□ml |
| 52 | Beer | □ | □/day | □/week | □/month | □□□ml |
| 53 | Wine | □ | □/day | □/week | □/month | □□□ml |
